# Thinking culture and critical thinking dispositions of high school students in Turkish Republic of Northern Cyprus

**DOI:** 10.3389/fpsyg.2022.1017747

**Published:** 2022-11-10

**Authors:** Tubanur Çelik İskifoğlu, Yaǧmur Çerkez, Gökhan İskifoğlu

**Affiliations:** ^1^Department of Psychological Counseling and Guidance, Near East University, Nicosia, Cyprus; ^2^Faculty of Education, European University of Lefka, Lefka, Turkey

**Keywords:** critical thinking, quantitative research, thinking culture, critical consciousness, family relationships

## Abstract

This study aimed to investigate the critical thinking disposition levels of the 15–18 age group of secondary and high school students in different educational settings in the Turkish Republic of Northern Cyprus (TRNC). Since culture is an inevitable part of thinking, this study becomes significant since there is no study investigating the thinking culture of youngsters in North Cyprus. After eliciting necessary permissions from the Ministry of Education and ethical boards, 1,130 participants in the age range of 15–18, who were selected by stratified random sampling and who voluntarily accepted to contribute to the study, took part as a targeted audience. Data was collected from six independent areas of North Cyprus. A Turkish version of the California Critical Thinking Disposition Inventory (CCTDI) was used as the main data collection tool and the data was collected *via* the online MS Teams platform. The study found that, first, most of the participants scored significantly below the desired criteria set by the related literature for the specified age group. Second, gender differences were studied, and girls were found to be more inclined to think critically than boys in terms of six facets of critical thinking except for truth-seeking. Third, an interesting result regarding urban-rural area distinction was elicited in favor of rural areas, which was contradicted by the related literature, and this finding is discussed under the cultural realms of Cyprus. The basic premises behind each finding and their causal associations with culture are elaborated in detail in the discussion section.

## Introduction

The demands and requirements of the 21st century require individuals to come up with more creative ideas and solutions to the problems of the era (Chou et al., 2019). Since globalization has so many expected and unexpected consequences on human life, new problems crop up every day requiring creative solutions. Since these problems are hardly local anymore, their solutions also need to be global. In such an era, which is characterized by fragmentation (Dumitru, 2019), it is becoming hard to define these problems and even harder to find solutions. Societies, therefore, need individuals who can face these challenges and yet be productive in supporting their country's economy through creative and innovative ideas (Hill et al., 2016). In this context, Hill et al. suggest that offering creative and unique ideas is somehow related to the thinking culture of societies. Thinking culture, in many studies, is defined as the way people think when they encounter a problematic situation (Andayani et al., 2020; Seibert, 2021). Formal education, on the other hand, is made of methodologies to shape people thinking in a certain way (Seibert, 2021). The former and the later arguments unpack the link between thinking culture and education. Some researchers underline the fact that the key to quality thinking is related to the quality of education a person receives (Fisher, 2007); the quality of the environment in which one is born and raised (Baker, 2013); the quality of family relationships (Brown, 1990); and the opportunities provided to individuals to establish a good state of health and psychological wellbeing (Arslan et al., 2014). An educational system that does not provide individuals with opportunities to practice the learned theory, experience knowledge with their five senses, and make inferences out of their experiences, cannot produce ideal individuals for societies (Abrami et al., 2008). The core element here is pragmaticism. A pragmatic educational system designs all input, processes, output, and evaluation in a way that students can play key roles in the design and production of education itself (Dewey, 1910). It is evident here that students are placed in the center of their own learning processes, and they are given more responsibilities for their own learning, which is where they begin to be more active rather than passive receivers of information. A decent education is characterized by the extent of thinking and practice opportunities given to students (Freire, 2005). All these arguments are about creating a thinking culture in society using education. As it is clear, the arguments are turning around the way education is provided to young people to create a better thinking culture within which individuals are creative.

A quality environment, which is also considered a part of the learning culture, is filled by people who are keen to support children and young people to achieve their goals and who are in a position of a critical observer who thinks and behaves as a model of a good problem solver (Pearl et al., 2019). Relatives, neighbors, and residents are considered significant individuals who are of great influence on the character of young people.

One other key component of thinking culture is family relationships. Every child, no doubt, wishes to be raised in a democratic and loving family. This is also a part of thinking culture. John Dewey, in his famous book “*Democracy and Education”*, asserts that the gate to a democratic society is held by dealing with the young (Dewey, 1916). It is such an important fact that our acts play an important role in their dispositions, which demonstrates that the quality of education given at home affects the way children think. Therefore, considering a child as a significant member of a family and considering their ideas as important as other members of a family have great consequences on the way these individuals think and behave when they become adults (Dewey, 1938). A recent study, which explored critical thinking in nursing students, pointed out that children who were raised in more democratic environments were found to be more inclined to think critically (Seibert, 2021).

Critical thinking abilities and dispositions, on the other hand, are such variables that begin to develop at younger ages through the development of the prefrontal cortex of the brain (Ku, 2009). It has taken a lot of effort to define critical thinking and its components. For instance, a two-year Delphi study, which involved 42 experts considered pioneers of the field, was conducted by the American Philosophical Association and supported by the California Academic Press yielded a consensus definition of critical thinking. According to the Delphi report, critical thinking disposition is known to be a characterological profile of a person that constitutes seven dispositional dimensions (Facione, 1990). The seven components of critical thinking dispositions are named as truth-seeking, open-mindedness, inquisitiveness, systematicity, analyticity, maturity of judgment, and critical thinking self-confidence (Facione, 1990). Although there are many studies about critical thinking and its correlates, very few of them have examined critical thinking at young ages (İskifoğlu, 2018). Critical thinking which is composed of two main dimensions as skills and dispositions have also subdimensions within each main dimension (Facione, 1990). Skills and dispositions are correlated in the sense that they support each other during the process of human cognition but are separated while forming ideas (Bailin et al., 1999). A great thinker and philosopher, John Dewey, explains this distinction in his famous book *How We Think*, by indicating that skills may either come as inborn or be developed later by practice, but disposition is a former entity that can only be developed by a human when necessary opportunities are provided, and it is for this reason that one should prefer to develop dispositions rather than skills to possess the positive inclination to develop thinking skills (Dewey, 1910). Unpacking this definition reveals that having a positive inclination toward critical thinking is a prerequisite for one to be creative in thinking, producing unique ideas, and using the skills of critical thinking. Seibert (2021) conducted a study to pinpoint the effect of some critical variables on critical thinking. One of the results suggested that it is getting even more difficult today to nurture generation Z's critical thinking dispositions with current methodologies. Unfortunately, there are many studies supporting the same result (Anazifa and Djukri, 2017; Basri, 2019; Andayani et al., 2020). The findings of these studies share the same notion that students' critical thinking levels are observed below their actual performances. Their main discussion point is related to the fact that students are not provided with enough opportunities to support their cognitive potential. The literature mentions the deficiencies but neither provides evidence for the causes nor displays results for young populations under the age of 18.

Against this context, it becomes important to shift attention to the critical thinking disposition levels of children who will engineer future societies. For this reason, studies are called to explore the extent to which children possess dispositions toward critical thinking in different educational settings (Facione, 2000). However, the majority of the studies concerning the issue of critical thinking concentrate on university-level students or older people (Cheung et al., 2001; İskifoğlu, 2018); an extensive review of the related literature confirms this. Very few studies concentrated on the thinking culture and critical thinking levels of young people under the age of 18 (Basri, 2019; Wan and Cheng, 2019; Seibert, 2021). Those studies were based on the views of individuals and carried out with interviews. No research existed exploring the issue from an empirical point of view for a valid inference regarding critical thinking as a part of thinking culture. However, a considerably high number of theoretical orientations have mentioned the importance of studying the thinking culture and critical thinking dispositions of people under the age of 18 since this demography is at a critical period of their cognitive development (Anazifa and Djukri, 2017). We have less information about how critical thinking is being processed at young ages with different educational systems in different settings in different societies. One of the most recent studies in the field explores critical thinking disposition at university-level students and most of the citations were mainly about nursing students and nursing education (Liu et al., 2021), which underlines the lack of related literature. Moreover, most recent studies showed that the dispositional dimension of critical thinking is best gained at young ages, especially at the 15–18 age period because the pre-frontal cortex of the brain, which is responsible for making inferences and judgments, begins to take its final development by the end of this age period (Moses and Baldwin, 2005; Ku, 2009; Wan and Cheng, 2019). Although brain development continues to take place in later periods of life, it will never ever be at the same flexible way or speed as it was at the 15–18 age period. For this reason, the lack of studies on younger populations has been criticized by pioneers in the field. Even though studies concerning younger populations for the last decade have started to deal with the situation from the perspective of younger generations, not enough empirical data exists to draw reliable generalizations and make invaluable inferences (Jaswal and Neely, 2006; Pasquini et al., 2007; Ku, 2009; Gibson, 2013; İskifoğlu, 2018). Since more studies need to be designed and conducted to fulfill the necessity to bring deeper insight into the problem, the current study aimed to explore the extent to which the 15–18 age group populations are disposed to think critically and to find out whether their levels of dispositions show significant differences in terms of several defined demographic variables.

The inference drawn from the review of the related literature led to paying attention to questioning the critical thinking disposition levels of the 15–18 age group high school students who are at the critical period where they will be establishing a level of disposition toward critical thinking. As mentioned earlier, the review of the related literature revealed that there is no study exploring the extent to which high school students are inclined to think critically and how their critical thinking levels were differentiated by some variables associated with established thinking culture. For this reason, the following research questions were addressed:

*Research Question 1: What are the critical thinking disposition levels of the 15–18 age group high school students as measured by CCTDI?*
*Research Question 2: Is there a significant difference between boys and girls in terms of the seven facets of critical thinking dispositions?*
*Research Question 3: Is there any significant difference between the regions where the participants live in terms of the seven facets of the CCTDI?*
*Research Question 4: Is there any significant difference between the socio-economic status of participants in terms of the seven facets of the CCTDI?*

## Methodology

### Research design

The current research, which employed a descriptive design, was supported by the quantitative research paradigm to explore the critical thinking dispositions of high school students regarding several critical exogenous variables. The rationale behind utilizing such a design is causally related to providing evidence if the observations obtained by the developed inventory are due to a bias or a true difference in the variables being measured (Anastasi and Urbina, 1997).

### Participants and data collection procedures

The first attempt in the data collection process was to get the necessary permissions from the board of ethics and the corresponding institutions. After eliciting necessary permissions from the Ministry of Education of TRNC to collect data, a highly representative sample was considered. A total of 3,722 participants from all over the secondary and high schools connected to the Ministry of Education of TRNC were selected by stratified random sampling method as a community sample. However, only 1,620 of them voluntarily accepted to participate in the study. After informing participants and their parents about their rights through informed consent forms and eliciting their consent, data collection procedures were started. As a result of the initial analysis, the distribution of participants according to the defined strata yielded that some participants should have been removed from the study to maintain a balanced categorical distribution. For that reason, 490 participants were removed from the necessary categories and 1,130 participants were left to continue with the analysis. Even though more than half of the participants either refused to participate or were eliminated for some statistical reasons, more than enough representative number of participants were available to contribute to the study.

The research sample included participants from the regions of Famagusta (*n* = 191, 16.9%), Nicosia (*n* = 214, 18.9%), Kyrenia (*n* = 199, 17.6%), Carpase (*n* = 176, 15.6%), Morphou (*n* = 177, 15.7%) and Lefka (*n* = 173, 15.3%) of Northern Cyprus. The age of the participants ranged from 15 to 18 with a mean age of 16.47 (SD = 1.12), and they were distributed as male (*n* = 566, 50.1%) and female (*n* = 564, 49.9%) in terms of gender.

### Data collection inventory

Based on the aim of this study, a Turkish version of the California Critical Thinking Disposition Inventory (CCTDI), which was first developed by Facione et al. (1992) from a detailed cross-disciplinary Delphi study and translated and adapted into Turkish language and culture by İskifoğlu (2014), was used to collect data from the targeted audiences. Brief definitions of the seven affective dispositions of the CCTDI are provided below (Facione et al., 1992, p. 11–12):

*Truth-seeking*: to “seek the truth, [be] courageous about asking questions, and [be] honest and objective about pursuing inquiry, even if the findings do not support one's interests or preconceived opinions.”
*Open-mindedness*: to be “open-minded and tolerant of divergent views with sensitivity to the possibility of one's own bias.”
*Analyticity*: to be “alert to potentially problematic situations, anticipating possible results or consequences, and prizing the application of reason and the use of evidence even if the problem at hand turns out to be challenging or difficult.”
*Systematicity*: to be “organized, orderly, focused, and diligent in inquiry.” Critical thinking self-confidence: “the level of trust one places in one's own reasoning processes.”
*Inquisitiveness*: to have “intellectual curiosity by means of valuing being well informed and learning, even if the immediate payoff is not directly evident.”
  *Maturity of judgment*: to make “reflective judgments based on cognitive maturity and epistemic development” (Facione et al., 1992, p. 11–12).

İskifoğlu (2014) reported that a series of nested model analyses resulted in a final model displaying acceptable reliability and validity for use with Turkish samples, χ^2^ = 730.35, df = 203, *p* < 0.001, χ^2^/df = 3.60; root mean square error of approximation (RMSEA) = 0.067, standardized root mean square residual (SRMR) = 0.06, comparative fit index (CFI) = 0.96. Iskifoglu also reported acceptable Cronbach's α for the subscales of the Turkish version of the CCTDI: truth-seeking (12 items), α = 0.85; open-mindedness (12 items), α = 0.82; analyticity (11 items), α = 90; systematicity (11 items), α = 0.86; inquisitiveness (10 items), α = 0.86; critical thinking self-confidence (nine items), α = 0.88; maturity of judgment (10 items), α = 0.81; and for the overall CCTDI (75 items), α = 0.87.

The scoring procedures of the CCTDI were not revealed by the copyright holders since the inventory is a copyrighted inventory. However, CCTDI scores are interpretive for each subscale. For example, scores for each subscale ranging between 10 and 29 are interpreted as a weak critical thinking disposition, scores ranging between 30 and 39 indicate an ambivalent critical thinking disposition, scores ranging between 40 and 49 indicate a positive critical thinking disposition and scores between 50 and 60 indicate a strong critical thinking disposition (İskifoğlu, 2014). Overall scores range between 70 and 420 and are interpreted based on the following standards: 70–209 signifies a negative disposition toward critical thinking, 210–279 signifies ambiguity or ambivalence toward critical thinking, and 280–420 signifies a positive disposition toward critical thinking (İskifoğlu, 2014).

### Data analysis procedures

Data collected from 1,130 participants *via* the Turkish version of the California Critical Thinking Disposition Inventory was analyzed through several statistical procedures using IBM-SPSS 24 software. First, the raw data was processed into more meaningful computable scores by means of data reduction procedures. The second step was to elicit the distributions and descriptive statistics regarding each exogenous and endogenous variable being studied. Especially, categorized variables were carefully detected by Kolmogorov Simirnov and Shapiro Wilk tests to get a deeper insight into whether the distributions of the data were normal across endogenous variables. The results of this preliminary analysis yielded that all endogenous variables were distributed normally across exogenous variables since all normality test results were found to be insignificant. Following that, each endogenous variable was tested across related exogenous variables to make inferences from the study. Since the study is based on a purely quantitative approach, no other analysis other than statistics was used.

## Results

*Research Question 1: What are the critical thinking disposition levels of the 15–18 age group high school students as measured by CCTDI?*

Table 1 lists the scores obtained for the various independent variables. According to those scoring procedures detailed earlier, it is evident that participants possess ambivalent disposition in terms of truth-seeking, open-mindedness, systematicity, inquisitiveness, and maturity of judgment; and they do display positive disposition in terms of critical thinking self-confidence, and analyticity (see Table 1). However, when overall scores were computed, it was found that their total inclination fell into the ambivalent category in general (see Table 1 for details).

**Table 1:** Descriptive statistics regarding independent variables being studied.

**Variables**	**N**	**Minimum**	**Maximum**	**Mean**	**Std. deviation**
TS	1,130	22.50	43.33	33.4167	4.89103
OPM	1,130	26.67	50.83	38.7603	4.97536
AN	1,130	26.36	50.91	40.6090	5.38688
SY	1,130	25.45	48.18	36.4360	4.40975
IN	1,129	28.00	48.00	38.8689	4.64355
CTS	1,130	27.78	56.67	42.6352	6.69742
MJ	1,130	19.00	50.00	34.3566	6.02234
TOTAL	1,130	217.07	313.48	265.0838	20.29948

TS, Truth-seeking; OPM, Open-mindedness; AN, Analyticity; SY, Systematicity; IN, Inquisitiveness; CTS, Critical thinking self-confidence; MJ, Maturity of judgment.

From the perspective of mean scores, the general picture was not quite clear in terms of data strata distributed for each category. So, in the main frame of the first research question, the categorized score groups were evaluated regarding their frequencies of occurrence. When frequencies for each category were computed, it was figured out that the majority of participants with high percentages fell into ambivalent and positive categories. Only a few participants were in the strong category for open-mindedness, analyticity, and maturity of judgment, except for critical thinking self-confidence. Unlike other dimensions, 105 (9.3%) participants tended to possess strong dispositions toward critical thinking self-confidence (see Table 2). It was also evident that none of the participants fell into the *strong* category for the facets of truth-seeking, systematicity, and inquisitiveness.

**Table 2:** Frequencies and percentages of distributions regarding dependent variables being studied.

**Variables**	**Weak *n* (%)**	**Ambivalent *n* (%)**	**Positive *n* (%)**	**Strong *n* (%)**
TS	306 (27.1)	671 (59.4)	153 (13.5)	-
OPM	38 (3.4)	572 (50.6)	507 (44.9)	13 (1.2)
AN	15 (1.3)	469 (41.5)	602 (53.3)	44 (3.9)
SYS	9 (0.8)	846 (74.9)	275 (24.3)	-
IN	44 (3.9)	531 (47.0)	555 (49.1)	-
CTS	37 (3.3)	274 (24.2)	714 (63.2)	105 (9.3)
MJ	238 (21.1)	659 (58.3)	226 (20)	7 (0.6)

TS, Truth-seeking; OPM, Open-mindedness; AN, Analyticity; SY, Systematicity; IN, Inquisitiveness; CTS, Critical thinking self-confidence; MJ, Maturity of judgment.

*Research Question 2: Is there a significant difference between boys and girls in terms of the seven facets of critical thinking dispositions?*

Gender is another dimension that is being argued in the field of critical thinking. So, it would have been remiss if gender were not studied in this research. Gender is an inevitable dimension of critical thinking studies. For this reason, several independent samples *t*-tests were conducted for each subscale of the CCTDI to determine if there are any gender differences in those facets. The results shockingly showed that except for the maturity of judgment scale, highly significant differences were apparent in the other remaining scales. Boys only performed significantly better than girls on the truth-seeking scale *t*(11.28) = −4.118, *p* < 0.001. On the other hand, girls performed significantly better than boys on the scales of open-mindedness *t*(11.28) = 4.574, *p* < 0.001; analyticity *t*(11.28) = 10.658, *p* < 0.001; systematicity *t*(11.28) = 5.722, *p* < 0.001; inquisitiveness *t*(11.28) = 4.417, *p* < 0.001; critical thinking self-confidence *t*(11.28) = 12.428, *p* < 0.001; and overall *t*(11.28) = 9.293, *p* < 0.001 (see Table 3 for means and standard deviations). When the table for descriptive statistics was checked, it is also evident that all mean scores were calculated with minimum acceptable errors.

**Table 3:** Descriptive statistics regarding dependent variables across gender.

**Variables**	**Gender**	**N**	**Mean**	**Std. deviation**	**Std. error mean**
TS	Girl	564	32.8206	4.91334	0.20689
	Boy	566	34.0106	4.79985	0.20175
OPM	Girl	564	39.4326	4.66890	0.19660
	Boy	566	38.0904	5.18096	0.21777
AN	Girl	564	42.2405	4.78142	0.20133
	Boy	566	38.9833	5.46808	0.22984
SY	Girl	564	37.1776	3.87836	0.16331
	Boy	566	35.6971	4.77210	0.20059
IN	Girl	563	39.4760	4.30228	0.18132
	Boy	566	38.2650	4.88926	0.20551
CTS	Girl	564	44.9626	6.62912	0.27914
	Boy	566	40.3161	5.92004	0.24884
MJ	Girl	564	34.3936	5.95194	0.25062
	Boy	566	34.3198	6.09673	0.25626
TOTAL	Girl	564	270.5045	19.19895	0.80842
	Boy	566	259.6822	19.93872	0.83809

TS, Truth-seeking; OPM, Open-mindedness; AN, Analyticity; SY, Systematicity; IN, Inquisitiveness; CTS, Critical thinking self-confidence; MJ, Maturity of judgment.

*Research Question 3: Is there any significant difference between the regions where the participants live in terms of the seven facets of the CCTDI?*

As it was continuously argued in the related literature, the characteristics of regions and environments in which children are raised are also considered to be influential on the quality of thinking (Aram and Aviram, 2009). Therefore, we examined if there was a significant difference between the defined areas (i.e., regions) where the participants lived in terms of their critical thinking dispositions. To address this question, one way analysis of variance (ANOVA) was used. The results of the analysis showed that participants' dispositions significantly differed according to their regions from the perspectives of truth-seeking f(5,1124) = 2.356, p>0.05 and systematicity f(5,1124) = 2.356, *p* > 0.05 (see Table 4 for details).

**Table 4:** Summary of results regarding ANOVA analysis of critical thinking disposition levels of participants in terms of their regions.

**Variables**		**Sum of squares**	**df**	**Mean square**	**F**	**Sig**.
TS	Between Groups	280.160	5	56.032	2.356	0.039
	Within Groups	26,727.965	1,124	23.779		
	Total	27,008.125	1,129			
OPM	Between Groups	149.198	5	29.840	1.207	0.304
	Within Groups	27,798.251	1,124	24.732		
	Total	27,947.449	1,129			
AN	Between Groups	251.119	5	50.224	1.736	0.123
	Within Groups	32,510.763	1,124	28.924		
	Total	32,761.882	1,129			
SY	Between Groups	233.641	5	46.728	2.418	0.034
	Within Groups	21,720.766	1,124	19.325		
	Total	21,954.406	1,129			
IN	Between Groups	208.283	5	41.657	1.940	0.085
	Within Groups	24,114.315	1,123	21.473		
	Total	24,322.599	1,128			
CTS	Between Groups	466.303	5	93.261	2.089	0.064
	Within Groups	50,175.541	1,124	44.640		
	Total	50,641.844	1,129			
MJ	Between Groups	367.466	5	73.493	2.036	0.071
	Within Groups	40,579.809	1124	36.103		
	Total	40,947.275	1129			
TOTAL	Between Groups	3,864.009	5	772.802	1.883	0.095
	Within Groups	461,361.632	1,124	410.464		
	Total	465,225.641	1,129			

TS, Truth-seeking; OPM, Open-mindedness; AN, Analyticity; SY, Systematicity; IN, Inquisitiveness; CTS, Critical thinking self-confidence; MJ, Maturity of judgment.

However, ANOVA did not show between which regions the true significant differences existed. For this reason, a follow-up advance analysis of group-wise *post hoc* test called LSD was conducted. The least significant difference (LSD) is one of the most conservative *post hoc* tests that can be applied to complex group-wise comparison analysis. The results yielded that the true difference existed between the Famagusta and Morphou regions in favor of the Morphou region (see Table 5) in terms of truth-seeking f(5,1124) = 2.356, *p* = 0.006 and between Nicosia and Cyrenia in favor of Cyrenia in terms of systematicity f(5,1124) = 2.418 *p* = 0.001 (see Table 5). No significant differences were apparent between other regions in terms of other facets. However, when Table 5 was carefully reviewed, it was evident that participants living in rural areas such as Morphou, Carpase, and Lefka performed better than participants living in urban areas such as Famagusta, Nicosia, and Cyrenia (see Table 5 for details).

**Table 5:** Descriptive statistics of dependent variables across defined regions.

**Variables**		**N**	**Mean**	**Std. deviation**	**Std. error**
TS	Famagusta	191	33.0497	4.54384	0.32878
	Nicosia	214	33.4502	4.93826	0.33757
	Cyrenia	199	33.0360	5.03901	0.35721
	Carpase	176	32.9877	4.97961	0.37535
	Morphou	177	34.4444	4.94498	0.37169
	Lefka	173	33.6031	4.78624	0.36389
	Total	1,130	33.4167	4.89103	0.14550
OPM	Famagusta	191	38.4642	5.37935	0.38924
	Nicosia	214	38.3762	4.89870	0.33487
	Cyrenia	199	38.8442	4.66213	0.33049
	Carpase	176	38.5227	5.04067	0.37995
	Morphou	177	39.0395	5.04591	0.37927
	Lefka	173	39.4220	4.79673	0.36469
	Total	1,130	38.7603	4.97536	0.14801
AN	Famagusta	191	40.5474	5.38785	0.38985
	Nicosia	214	39.7621	5.51304	0.37686
	Cyrenia	199	40.9959	5.09251	0.36100
	Carpase	176	41.0589	5.46761	0.41214
	Morphou	177	40.4366	5.63084	0.42324
	Lefka	173	40.9984	5.15721	0.39210
	Total	1,130	40.6090	5.38688	0.16025
SY	Famagusta	191	36.3732	4.41535	0.31948
	Nicosia	214	35.7009	4.06476	0.27786
	Cyrenia	199	37.1402	4.50059	0.31904
	Carpase	176	36.6012	4.51260	0.34015
	Morphou	177	36.2404	4.27652	0.32144
	Lefka	173	36.6369	4.64084	0.35284
	Total	1,130	36.4360	4.40975	0.13118
IN	Famagusta	191	39.1518	4.58062	0.33144
	Nicosia	214	38.2617	4.87754	0.33342
	Cyrenia	199	39.3970	4.08844	0.28982
	Carpase	176	39.2159	4.62527	0.34864
	Morphou	177	38.4124	4.81174	0.36167
	Lefka	172	38.8140	4.79525	0.36563
	Total	1,129	38.8689	4.64355	0.13820
CTS	Famagusta	191	43.4380	6.96893	0.50425
	Nicosia	214	42.0197	6.49398	0.44392
	Cyrenia	199	42.6857	7.01235	0.49709
	Carpase	176	43.4154	6.43515	0.48507
	Morphou	177	41.7012	6.56385	0.49337
	Lefka	173	42.6140	6.55893	0.49867
	Total	1,130	42.6352	6.69742	0.19924
MJ	Famagusta	191	33.8168	6.12097	0.44290
	Nicosia	214	34.1495	5.76912	0.39437
	Cyrenia	199	34.8090	6.17291	0.43759
	Carpase	176	33.4943	6.09918	0.45974
	Morphou	177	34.8249	5.75028	0.43222
	Lefka	173	35.0867	6.14633	0.46730
	Total	1,130	34.3566	6.02234	0.17915
TOTAL	Famagusta	191	264.8411	21.83828	1.58016
	Nicosia	214	261.7203	20.32934	1.38969
	Cyrenia	199	266.9081	19.19889	1.36097
	Carpase	176	265.2962	20.12548	1.51701
	Morphou	177	265.0994	19.73003	1.48300
	Lefka	173	267.1819	20.22316	1.53754
	Total	1,130	265.0838	20.29948	0.60387

TS, Truth-seeking; OPM, Open-mindedness; AN, Analyticity; SY, Systematicity; IN, Inquisitiveness; CTS, Critical thinking self-confidence; MJ, Maturity of judgment.

*Research Question 4: Is there any significant difference between the socio-economic status of participants in terms of the seven facets of the CCTDI?*

As the last concern of this study, we investigated if participants belonging to different socio-economic statuses significantly differentiate regarding the seven dispositional attributes of critical thinking. One-way analysis of variance results yielded that significant differences existed for all the facets of the CCTDI across socio-economic categories (see Table 7). The LSD *post hoc* test was conducted to bring deeper insight into where the differences existed between each category. When the pairwise LSD *post hoc* outputs were evaluated, the results outlined that participants who belonged to high socio-economic status (between 4000 and 5000 TL) performed better than participants who belonged to other three lower socio-economic levels f(5,1124) = 28.127 *p* = 0.010 for truth-seeking subscale (see Table 6 for means and standard deviations and Table 7 for ANOVA statistics). On the other hand, participants who were in category four performed significantly better than all other lower categories in terms of open-mindedness f(5,1124) = 1,666.403 *p* = 0.000 (see Table 6 for means and standard deviations and Table 7 for ANOVA statistics).

**Table 6:** Descriptive statistics of dependent variables regarding socio-economic status of participants.

**Variables**		**N**	**Mean**	**Std. deviation**	**Std. error**
TS	Cat 1. Lower than 2000 TL	136	32.4449	5.09196	0.43663
	Cat 2. Between 2000TL−3000TL	273	32.2863	4.97185	0.30091
	Cat 3. Between 3000TL−4000TL	288	33.6863	4.96219	0.29240
	Cat 4. Between 4000TL−5000TL	198	36.3973	4.36754	0.31039
	Cat 5. Between 5000TL−7000TL	184	31.6621	3.38585	0.24961
	Cat 6. Above 7000TL	51	35.2941	4.14268	0.58009
OPM	Cat 1. Lower than 2000 TL	136	41.3174	5.19135	0.44515
	Cat 2. Between 2000TL−3000TL	273	38.5836	4.47378	0.27077
	Cat 3. Between 3000TL−4000TL	288	34.9855	4.35462	0.25660
	Cat 4. Between 4000TL−5000TL	198	42.8577	3.22940	0.22950
	Cat 5. Between 5000TL−7000TL	184	38.5915	3.29747	0.24309
	Cat 6. Above 7000TL	51	38.9052	4.57496	0.64062
AN	Cat 1. Lower than 2000 TL	136	44.4652	3.99950	0.34295
	Cat 2. Between 2000TL−3000TL	273	40.5961	5.36174	0.32451
	Cat 3. Between 3000TL−4000TL	288	39.1004	5.13614	0.30265
	Cat 4. Between 4000TL−5000TL	198	45.8007	5.09535	0.36211
	Cat 5. Between 5000TL−7000TL	184	46.0119	3.92577	0.28941
	Cat 6. Above 7000TL	51	47.1676	6.59154	0.92300
SY	Cat 1. Lower than 2000 TL	136	37.0722	3.31157	0.28397
	Cat 2. Between 2000TL−3000TL	273	37.1096	4.22112	0.25547
	Cat 3. Between 3000TL−4000TL	288	35.1452	3.75198	0.22109
	Cat 4. Between 4000TL−5000TL	198	36.8916	5.50591	0.39129
	Cat 5. Between 5000TL−7000TL	184	48.4130	3.82252	0.28180
	Cat 6. Above 7000TL	51	36.7380	6.68828	0.93655
IN	Cat 1. Lower than 2000 TL	136	41.4926	3.22260	0.27634
	Cat 2. Between 2000TL−3000TL	273	38.7656	4.50938	0.27292
	Cat 3. Between 3000TL−4000TL	288	39.5764	4.81227	0.28357
	Cat 4. Between 4000TL−5000TL	198	42.7828	5.87849	0.41777
	Cat 5. Between 5000TL−7000TL	183	37.5410	2.84532	0.21033
	Cat 6. Above 7000TL	51	37.4118	3.77982	0.52928
CTS	Cat 1. Lower than 2000 TL	136	44.1095	7.46560	0.64017
	Cat 2. Between 2000TL−3000TL	273	42.1001	5.30852	0.32129
	Cat 3. Between 3000TL−4000TL	288	42.5386	6.47905	0.38178
	Cat 4. Between 4000TL−5000TL	198	49.5612	6.26817	0.44546
	Cat 5. Between 5000TL−7000TL	184	45.3140	7.54813	0.55646
	Cat 6. Above 7000TL	51	40.5011	7.01080	0.98171
MJ	Cat 1. Lower than 2000 TL	136	34.6103	7.93881	0.68075
	Cat 2. Between 2000TL−3000TL	273	35.1282	5.70813	0.34547
	Cat 3. Between 3000TL−4000TL	288	31.7465	4.88134	0.28764
	Cat 4. Between 4000TL−5000TL	198	39.4798	3.86013	0.27433
	Cat 5. Between 5000TL−7000TL	184	32.1630	4.89737	0.36104
	Cat 6. Above 7000TL	51	36.1961	7.55783	1.05831
Total	Cat 1. Lower than 2000 TL	136	275.5121	22.06562	1.89211
	Cat 2. Between 2000TL−3000TL	273	264.5695	20.16206	1.22026
	Cat 3. Between 3000TL−4000TL	288	256.7790	16.90253	0.99599
	Cat 4. Between 4000TL−5000TL	198	275.7712	18.40816	1.30821
	Cat 5. Between 5000TL−7000TL	184	260.7099	16.66751	1.22875
	Cat 6. Above 7000TL	51	261.2138	23.54192	3.29653

TS, Truth-seeking; OPM, Open-mindedness; AN, Analyticity; SY, Systematicity; IN, Inquisitiveness; CTS, Critical thinking self-confidence; MJ, Maturity of judgment; TL, Turkish Lira; Cat, Category.

**Table 7:** Summary of results regarding ANOVA analysis of critical thinking disposition levels of participants in terms of their socio-economic status.

**Variables**		**Sum of squares**	**df**	**Mean square**	**F**	**Sig**.
TS	Between Groups	3,003.451	5	600.690	28.127	0.000
	Within Groups	24,004.674	1,124	21.356		
	Total	27,008.125	1,129			
OPM	Between Groups	8,332.014	5	1,666.403	95.488	0.000
	Within Groups	19,615.435	1,124	17.451		
	Total	27,947.449	1,129			
AN	Between Groups	51,04.455	5	1,020.891	41.489	0.000
	Within Groups	27,657.427	1,124	24.606		
	Total	32,761.882	1,129			
SY	Between Groups	704.607	5	140.921	7.454	0.000
	Within Groups	21,249.799	1,124	18.906		
	Total	21,954.406	1,129			
IN	Between Groups	1,747.833	5	349.567	17.389	0.000
	Within Groups	22,574.766	1,123	20.102		
	Total	24,322.599	1,128			
CTS	Between Groups	2,780.829	5	556.166	13.061	0.000
	Within Groups	47,861.015	1,124	42.581		
	Total	50,641.844	1,129			
MJ	Between Groups	6,557.353	5	1,311.471	42.864	0.000
	Within Groups	34,389.922	1,124	30.596		
	Total	40,947.275	1129			
TOTAL	Between Groups	61,625.297	5	12,325.059	34.324	0.000
	Within Groups	403,600.344	1,124	359.075		
	Total	465,225.641	1,129			

TS, Truth-seeking; OPM, Open-mindedness; AN, Analyticity; SY, Systematicity; IN, Inquisitiveness; CTS, Critical thinking self-confidence; MJ, Maturity of judgment.

Participants who are in category four performed significantly higher than all lower socio-economic levels in each category for analyticity f(5,1124) = 41.489 *p* = 0.000, for systematicity f(5,1124) = 7.454 *p* = 0.001, for inquisitiveness f(5,1124) = 349.567 *p* = 0.001, for critical thinking self-confidence f(5,1124) = 556.166 *p* = 0.000, for maturity of judgement f(5,1124) = 1,311.471 *p* = 0.000 and for overall inclination scores f(5,1124) = 12,325.59 *p* = 0.000 (see Table 6 for means and standard deviations and Table 7 for ANOVA statistics). What is interesting in those results is that no significant difference was observed between category 6 and other previous categories in terms of all sub-dimensions of critical thinking disposition levels of the participants.

## Discussion

Critical thinking disposition, which is considered a critical characterological profile of a productive thinker (Dewey, 1910; Facione, 1990; Bailin et al., 1999), is an inevitable necessity that one must hold to overcome the challenges of the 21^st^ century. A considerable body of evidence insistingly mentions the importance of this human dimension as a major concern of human sciences (İskifoğlu, 2014, 2018; Terblanche and de Clercq, 2019; Wan and Cheng, 2019). Cognitive philosophers and scholars who follow the ism of Dewey also hold educational systems responsible for nurturing critical thinking dispositions of individuals at younger ages. Yet, many studies show that current young generations do not hold sufficient levels of inclination to critical thinking (Cheung et al., 2001; Arslan et al., 2014). Among many reasons, family socio-economic background, social environment, quality of education, and culture have been shown as the primary root cause of the problem (Gee and Heyman, 2007; Gibson, 2013; Günay and Çarikçi, 2018). For the sake of defining each of these causes for the problem, studies accelerated to describe each problem and to find out practical solutions. However, most of the studies worked on either university-level populations or older. Yet very few studies concentrated on younger populations (Gee and Heyman, 2007; Gibson, 2013; Günay and Çarikçi, 2018).

Several descriptive analyses and inferential statistical analyses were run for each independent variable based on each dependent variable. In order not to repeat the same procedures, we better prefer to summarize, criticize, and draw some inferences based on the results of this study in light of the related literature.

The first remark to make about the results is that a significant number of students displayed insufficient disposition in all the seven facets of critical thinking. Only one-third of the participants displayed a positive disposition. A significantly and exceptionally low number of participants displayed a strong disposition toward open-mindedness, analyticity, critical thinking self-confidence, and overall scale. In addition to that, no participant displayed a strong disposition toward truth-seeking, systematicity, inquisitiveness, and maturity of judgment (see Tables 1, 2). Likewise, this evidence has been drawn from a significantly high and representative number of participants from TRNC that covers all areas of the region. Yet, not this level of low scores was expected from participants. Unexpectedly, a majority of the participants scored quite below the acceptable scores (see Methodology section for acceptable scores) defined by the related literature (İskifoğlu, 2014). The related literature supports this finding. For instance, the study of Günay and Çarikçi (2018), which was conducted in a similar context in Turkey with young learners under the age of 18 and explored critical thinking levels revealed that the observed levels of critical thinking were observed to be far below the critical level. Gunay and Çarikçi linked the reason for this problem to the structure of the educational system, which mainly concentrates on memorization and retention rather than analysis and evaluation. The Turkish Republic of Northern Cyprus is no different from Turkey in terms of its educational system and students start to get used to multiple-choice-questions exam format in a memorization-oriented educational system. As they progress through the levels of education, the need to memorize information increases, which creates an unexpected chain reaction.

Critical pedagogy, an approach to systematize education in a way that caters to the needs of the defined age group to nurture critical thinking dispositions has always been mentioned as a key to solving problems (Freire, 2005). Though, this was not a solution in the TRNC case. For that matter, we sought out the root causes to identify the problems from other perspectives. The first step was to check if there was a difference between boys and girls in terms of the seven dispositional facets of critical thinking and we discovered that girls performed significantly much better than boys in most of the facets except for truth-seeking. A similar finding was observed in a recent study conducted in TRNC and girls' performances on reading skills were found to be better than boys (Koç, 2020). Koç asserted that reading skills are also associated with critical thinking. One other study conducted by Karahan and Iskifoglu indicated that there is a strong correlation between reading skills and critical thinking dispositions of students at the university level (Karahan and Iskifoglu, 2020). These recent studies support our finding although they are not carried out with young children. Yet more explanations for the cause of this finding are required. A plausible explanation for this finding forwarded by a study that explored gender differences in critical thinking is that boys in Turkish culture were raised with more freedom of social interaction whereas girls' movements and social moratorium chances were limited because of cultural realms, which in turn led girls to use more of their critical consciousness than boys (Hindman et al., 2008; Günay and Çarikçi, 2018). Because boys did not feel any necessity to create solutions and because they have not encountered social pressure, they did not need to use critical consciousness to solve such social problems in comparison to girls.

Another perspective of the current research included the distinctions between areas in which participants live. According to the explanations proposed by some researchers, individuals living in more rural areas tend to develop less disposition toward critical thinking because of fewer social opportunities they have in comparison to those who live in urban areas (Aram and Aviram, 2009; Baker, 2013; Gibson, 2013; Arslan et al., 2014). However, the results regarding this in the current study are in contradiction with the literature. Not only did the participants who live in rural areas perform better in general but there were significant differences in most cases in favor of rural areas in comparison to urban residents. Similar to the contradiction in the gender-related observation, here is a dilemma with the literature. So, this finding attracted our attention to rethink about the rural and urban area distinctions in favor of urban areas, which is usually accepted as a rule of thumb in most outlets.

Socio-economic status was one of the most discussed variables which were continuously associated with high critical thinking dispositions. As the last concern of this research, we investigated this variable in terms of seven facets of critical thinking dispositions. The interpretation of the results showed similar inferences in line with the related literature that the higher the socio-economic status, the higher the critical thinking dispositions (Gibson, 2013). Although socio-economic status has been considered a determinant factor for better critical thinking, some other studies argue that there might be a hidden variable between socio-economic status and critical thinking. For instance, a very recent study conducted by Seibert (2021) mentions the *appropriate use of opportunities* to foster critical thinking. As Seibert puts it, if these opportunities are provided by practitioners at schools, then, individuals from low socio-economic status may benefit from the results of these opportunities.

## Conclusion

In conclusion, as a matter of Occam's razor, the most striking point is the fact that our young students in the age range of 15–18, studying at secondary and high schools in different regions of the TRNC do not have a sufficient inclination toward critical thinking, which theoretically means that they are not going be able to use their thinking skills to produce unique ideas and find creative solutions for the problems they will encounter in their life. Especially when we consider the value given to critical thinking to survive in the system by significant scholars, one cannot help but ask: what is the reason behind the unexpectedly low performances of young individuals who are at their critical age periods to develop a positive characterological profile of critical thinkers? And how can we bring such a change into being that turns the situation the other way around so that more productive individuals are embedded into our society? We know that every dramatic improvement requires political decisions for reformed educational policies because it is quite visible that the quality of education individuals receive is a big determinant factor. Finally, just like everything about human behavior that is explained by culture, critical thinking is no exception and should be studied from the perspective of culture. Countries that determine their furthest goals, general goals, and specific goals on the basis of cognitive, affective, and psychomotor domains in terms of the needs of the country with a precise intention to produce effective individuals will be more successful in terms of economic realms and individual life expectations.

## Recommendations and future implications

Culture may be an explanation for these findings, but is not enough to cover the issue since such a complex matter cannot be explained by one dimension. Therefore, more, and further studies need to be conducted, especially regression-oriented studies, to investigate possible latent associations regarding these findings.

Gender studies are not only constituted to extract meanings from a perspective of gender roles, but it also leans toward other invisible dimensions such as other culture-oriented dimensions. Then, this can be studied with a detailed qualitative approach to bring deeper insight into why girls performed better than boys in terms of critical thinking dispositions.

Participants in rural areas performed better than participants in urban areas in the critical thinking disposition test. Why did participants living in rural areas perform better? This may be related to the understanding or labeling of areas as rural or urban. Today, it is not as easy as it was a decade ago to make a distinction between the concepts of rural areas and urban areas. This is actually because of globalization. Globalization created a world order and economic situation where everything can be everywhere at any given time. This study lacks for explaining the reason behind this finding. Not a fluke or a coincidence but true evidence showed that this is a fruitful area to study. What was unique to our study was that the participants were composed of children between the ages of 15 and 18, and there are very few studies that have concentrated on that age group. Besides, those studies were all qualitative rather than empirical quantitative studies. Therefore, the same study can be conducted with similar participants in different educational contexts in different countries across the world to create cross-cultural comparative studies.

## Data availability statement

The raw data supporting the conclusions of this article will be made available by the authors, without undue reservation.

## Ethics statement

The studies involving human participants were reviewed and approved by Ethical Committee Board of Near East University of Lefka. Written informed consent to participate in this study was provided by the participants' legal guardian/next of kin.

## Author contributions

All authors listed have made a substantial, direct, and intellectual contribution to the work and approved it for publication.

## Conflict of interest

The authors declare that the research was conducted in the absence of any commercial or financial relationships that could be construed as a potential conflict of interest.

## Publisher's note

All claims expressed in this article are solely those of the authors and do not necessarily represent those of their affiliated organizations, or those of the publisher, the editors and the reviewers. Any product that may be evaluated in this article, or claim that may be made by its manufacturer, is not guaranteed or endorsed by the publisher.
